# Study on the in vitro digestion process of green wheat protein: Structure characterization and product analysis

**DOI:** 10.1002/fsn3.2947

**Published:** 2022-06-03

**Authors:** Kangyi Zhang, Qingyu Wen, Yufei Wang, Tianqi Li, Bin Nie, Yu Zhang

**Affiliations:** ^1^ Center of Agricultural Products Processing Henan Academy of Agricultural Sciences Zhengzhou China; ^2^ Henan International Joint Laboratory of Whole Grain Wheat Products Processing Zhengzhou China; ^3^ Henan Province Whole Grain Fresh Food Processing Engineering Technology Research Center Zhengzhou China; ^4^ School of Chemical Engineering and Technology North University of China Taiyuan China; ^5^ Henan Ankang Food Science and Technology Research Institute Zhengzhou China; ^6^ Henan Ankang Future Food Technology Co., Ltd Zhengzhou China; ^7^ Resource Utilization Department of Henan Provincial Department of Agriculture and Rural Zhengzhou China; ^8^ College of Food Science and Nutritional Engineering China Agricultural University Beijing China

**Keywords:** digestion products composition, green wheat protein, in vitro digestion, structure

## Abstract

In this study, the in vitro digestion process of green wheat protein (GWP) was explored by simulating the gastrointestinal digestion. The digestibility of GWP was 65.23%, and was mainly digested by trypsin. During the digestion process of GWP, large‐size particles are digested by pepsin, and medium‐sized particles are digested by trypsin into smaller particles; irregular large block structure with smooth surface was gradually turned into smaller blocks with porous surface; and the spatial conformation was loosened mainly by the unfolding of β‐sheet structure. Gel electrophoresis demonstrated that HMW glutenin and ω‐gliadins in GWP were completely digested, while LMW glutenin and α/β/γ‐gliadins were partially digested. Additionally, the peptide lengths were relatively dispersed after pepsin digestion. Most of the peptides (76.5%) fell into the range 3–15 amino acid after pepsin and trypsin digestion. The molecular weight (MW) of most pepsin digestion products was above 2000 Da, whereas the MW of trypsin digestion products was mainly concentrated in 500–2000 Da. Besides, the sensitizing peptide sequence of wheat protein was detected in the final digestion products of GWP. This research provided a theoretical guidance for the development and application of GWP.

## Graphical abstract

Digestion process of green wheat protein was systematically characterized. Peptides of in vitro digestion products were distributed in ranges of 500‐2000 Da. Immunogenic peptides causing celiac disease were presenced in digestion products.

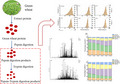

## 1 INTRODUCTION

As plant proteins are increasingly used as a source of amino acids in the diet (Carla et al., 2021), studies on in vitro digestion of plant proteins are key to understand the different factors affecting proteolysis, with the ultimate goal of optimizing the nutritional composition/intake of plant protein‐rich products (Torcello‐Gómez et al., 2020). Green wheat is a plump but immature wheat grain with a green color and unique smoky flavor. The best time for harvesting green wheat is when some milky endosperm exudes from a grain bent sharply between thumb and forefinger (Al‐Mahasneh & Rababah, 2007). The content of protein and dietary fiber in wheat is higher during this period. It was reported that the protein content of green wheat is 14.9%, while this content is 11.9% in mature wheat (Zhang & Zhang, 2019). In addition, the mineral composition of green wheat shows high amounts of potassium, calcium, and magnesium (Zhang, Yang, et al., 2020). It is worth noting that the higher content of dietary fiber in green wheat is beneficial to human intestinal digestion and detoxification. Green wheat is recognized as a healthy food because of its high nutritional value, unique flavor (Zhang et al., 2021), and its ability to help the body digest and stabilize blood sugar (Zhang, Zhang, et al., 2020). At present, it has been used as a food raw material to make cakes, bread, noodles, steamed buns, fried dough sticks, Nianzhuan, Zongzi, health drinks, etc. (Kang et al., 2015; Kang et al., 2017). As a fresh whole grain food, green wheat has a broad market prospect and economic value. With the improvement of living standards and the diversified development of food consumption, more and more attention is paid to the nutritional properties of the food while pursuing food flavor. In terms of green wheat food, protein is its main nutrient. Although the protein content of green wheat is high, the degree of digestion and absorption by the body is the key to determining its nutritional value. However, the digestive properties of green wheat protein (GWP) have not been systematically studied.

In the study of food digestion characteristics, the in vitro digestion model is a crucial research method (Rengadu et al., 2020). A two‐step digestion method based on pepsin and trypsin is widely employed to simulate the food digestion process in the stomach and small intestine (Villas‐Boas et al., 2015). As an example, pepsin and trypsin have been used to conduct a simulated in vitro digestion study on the protein in dry‐cured ham from different regions. The digestibility and particle size of ham in different regions were significantly varied during the digestion process (Jiang et al., 2019). In addition, increasing researchers have begun to focus on the compositions of protein digestion products to evaluate the nutritional value of protein. In the research of in vitro digestion of defatted walnut meal protein, it was found that polypeptides of molecular weight (MW) < 3000 Da (21.66 mg/ml) and more free amino acids (FAA) (8.09 mg/ml) were observed in the digested products. What is more, the digested products generated high angiotensin‐I‐converting enzyme (ACE) inhibitory activity (42.9%) and DPPH free radical scavenging activity (Liu et al., 2019). Although there have been many studies on in vitro digestion of protein, there is no exploration of the in vitro digestion process of protein extracted from green wheat. As a whole grain, green wheat meets people's needs for health and nutrition. Its nutritional value characterization is essential for its development and application.

Therefore, the purpose of this study is to clarify the digestion process of GWP and the composition of digestion products. Structural changes in protein during the digestion process were characterized using spectroscopic experiments and scanning electron microscope (SEM) techniques. Sodium dodecyl sulfate polyacrylamide gel electrophoresis (SDS‐PAGE) and liquid chromatograph‐mass spectrometer (LC‐MS) were applied to investigate the alteration of MW distribution during the digestion process, and analyze the composition of the digestion products. This research attempts to systematically characterize the digestive properties of GWP to provide a theoretical basis for the development and application of green wheat.

## 2 MATERIAL AND METHODS

### 2.1 Materials

Green wheat (Bainong 201) was provided by Henan Academy of Agricultural Sciences. Standard protein marker (15 kDa to 99 kDa) was purchased from Solarbio Technology (Beijing, China). Pepsin (3200 U/mg), trypsin (4 U/mg), and chromatographic grade trifluoroacetic acid (TFA) were purchased from Sigma‐Aldrich Co. (St. Louis, MO, USA). All other chemicals were of analytical grade.

### 2.2 Preparation of green wheat protein

GWP was prepared according to the reported method with slight modification (Hettiarachchy et al., 1996). Green wheat grains were prefrozen at −40°C, and freeze dried in a freeze dryer (FD‐100S, Beijing Huicheng Jiayi Technology Co., Ltd.), then the freeze drying was completed until the temperature rose to around 25–30°C. The freeze‐dried green wheat was pulverized into powder, and then passed through a 60 mesh sieve. A certain amount of green wheat flour and chloroform were mixed at a ratio of 1:2 (w/v) and stirred for 5 min, and the suspension was filtered with a Buchner funnel. This procedure was repeated three times for the remaining green wheat flour. Defatted green wheat flour was placed in a fume hood to let it dry naturally at room temperature until there was no chloroform smell.

Defatted green wheat flour was mixed with distilled water at a ratio of 1:15 (w/v) and stirred for 30 min. Its pH was adjusted to 11 with 1 mol/L NaOH, followed by stirring at 50°C for 1.5 h. The mixture was centrifuged at 4000 r/min for 15 min to collect the supernatant. The residue was extracted again and the supernatant was combined with that collected for the first time, and the remaining residue was discarded. The pH value of the supernatant was adjusted to the isoelectric point of protein (pH 4.7) with 1.0 mol/L HCL. It was then stirred at room temperature for 30 min and stood for 20 min. The precipitate was collected after centrifugation at 4000 r/min for 15 min. GWP was obtained after freeze drying. The content of GWP was about 91% after being determined by Kjeldahl nitrogen.

### 2.3 In vitro digestion

INFOGEST in vitro simulated digestive system was used to digest GWP, including two stages of stomach and intestine (Brodkorb et al., 2019). The preparation of simulated gastric fluid (SGF) and simulated intestinal fluid (SIF) were shown in Table 1. Digestive juices of SGF (200 ml) were composed of 1.2 g pepsin, 150 ml SGF, 0.1 ml CaCl(H_2_O)_2_, and deionized water, then the pH of the mixed solution was adjusted to 2.0. Moreover, 0.16 g trypsin and 1.70 g bile salt were weighed and mixed with 150 ml SIF, 0.4 ml CaCl(H_2_O)_2_ (0.3 mol/L) was added and diluted to 200 ml, then the pH was adjusted to 7.0 to prepare a digestion solution of SIF.

**TABLE 1:** Preparation of simulated digestion fluids

Composition	Solution concentration		SGF		SIF	
	g/L	mol/L	Volume/ml	Concentration (mmol/L)	Volume/ml	Concentration (mmol/L)
KCl	37.3	0.5	6.9	6.9	6.8	6.8
KH_2_PO_4_	68.0	0.5	0.9	0.9	0.8	0.8
NaHCO_3_	84.0	1.0	12.5	25.0	42.5	85.0
NaCl	117.0	2.0	11.8	47.2	9.6	38.4
MgCl(H_2_O)_6_	30.5	0.15	0.4	0.1	1.1	0.33
(NH_4_)_2_CO_3_	48.0	0.5	0.5	0.5	–	–
CaCl(H_2_O)_2_	44.1	0.3	–	0.15	–	0.6

*Note*: The final volumes of SGF and SIF are both 500 ml.

GWP was dissolved in deionized water (0.5%, M/V), and an equal volume of digestive juices of SGF was preheated in a 37 °C water bath for 5 min; mixed well and shaken at 37 °C constant temperature (120 r/min) for 2 h. Samples were taken at 10, 20, 30, 60, 90, and 120 min of digestion time. The digestive juice after gastric digestion was adjusted to pH of 7.6 with 1 mol/L NaOH, and an equal volume of digestion solution of SIF (37 °C) was added. Then, the mixed solution was oscillated at 37 °C constant temperature (120 r/min) for 2 h to complete the digestion. The sampling time was 10, 20, 30, 60, 90, and 120 min. After the simulated digestion was completed, the enzyme was inactivated in a boiling water bath (5 min), and the pH was adjusted to 7.0. Then, digestion products were centrifuged at 11,000 r/min for 15 min, the supernatant was frozen at a low temperature, and the precipitate was lyophilized for further use.

### 2.4 In vitro digestibility

A certain amount of gastric digestive fluid, intestinal digestive fluid, and undigested protein samples were evenly mixed with 10% trichloroacetic acid (TCA), respectively, and then centrifuged at 8000 r/min for 15 min. Kjeldahl's method was employed to determine the protein contents of the samples (Klepper & Wilhelmi, 1979), and the in vitro digestibility of the samples was calculated in the following formula:

(1) $$\text{In vitro digestibity}\;\left(\%\right)=\frac{\text{Protein content before digestion}‐\text{Protein content after digestion}}{\text{Protein content before digestion}}.$$

### 2.5 Particle size distribution

Particle size distribution of GWP before and after digestion was detected by a laser particle size analyzer (Sync, Microtrac Inc., USA). The GWP samples were scanned with the relative refractive index set to 1.54 and absorption rate set to 0.001. Each sample was scanned three times.

### 2.6 Scanning electron microscopy (SEM)

Sample microstructure changes were observed using a scanning electron microscope (SEM) (Hitachi S‐3400, Japan). The samples that had passed through a 100‐mesh sieve were evenly spread at a layer on double‐sided tape and placed on a gold plate for vacuum plating. In the low vacuum mode under the voltage of 20 kV, the surface morphology of the samples at 1000 and 5000 times was observed by SEM.

### 2.7 Intrinsic fluorescence spectroscopy

Intrinsic fluorescence spectroscopy of the samples was detected with a fluorescence spectrophotometer (Agilent Co. Ltd., CA, USA). The samples were diluted to a certain concentration with phosphate buffer solution (PBS). The fluorescence spectrum was scanned under the excitation wavelength 280 nm, emission wavelength 290–540 nm, and slit width 5 nm.

### 2.8 Fourier transform infrared (FTIR) spectroscopy

FTIR spectroscopy was detected using an infrared spectrometer (Thermo Fisher Scientific, Waltham, MA, USA). Samples of freeze‐dried powder and potassium bromide (KBr) were thoroughly mixed at a ratio of 1:150, ground into a fine powder, and compressed into thin slices. FTIR spectrum was scanned from 400 cm^−1^ to 4000 cm^−1^ wavelengths with 4 cm^−1^ resolution, and the number of scans was 64. Each sample was scanned in three replicate. Peak Fit software V4.12 was used to perform peak fitting and quantitative analysis of the secondary structure in the amide I region.

### 2.9 SDS‐PAGE

The SDS‐PAGE method was slightly modified according to the reported method (Lin et al., 2020). GWP and its digestion products at different times were dissolved into 500 μl distilled water. The suspension (30 μl) and loading buffer (10 μl) were mixed and shaken for 10 min. The samples were kept in a water bath at around 100 °C for 8 min, and centrifuged at 10,000 r/min for 10 min after cooling. The concentrations of the separating gel and stacking gel were 12% and 5%, respectively. In addition, the loading volume was 15 μl, and the voltages of the stacking gel and separating gel were set to 80 V and 150 V, respectively. Electrophoresis was completed when bromophenol blue moved to the bottom of the gel. The gel was stained for 20 min with Coomassie Brilliant Blue R‐250 staining solution, and then put into a decolorizing solution for decolorization until transparency. The MW marker of 15–99 kDa was used to estimate the MW of the samples.

### 2.10 High‐performance liquid chromatography (HPLC)

The MW distribution of the digestion products was determined according to the reported method with minor changes (Jiang et al., 2018). HPLC 1260 (Agilent Co. Ltd., California, USA) and Protein‐Pak 60 (7.8 mm × 300 mm, Waters) were selected for this experiment. The concentration of the samples was 1 mg/ml. The diluted samples were filtered through 0.45 μm filter membrane and then injected into a 2 ml injection vial. The mobile phase included 20% acetonitrile, 0.1% TFA, and ultrapure water. The column was equilibrated and eluted at a flow rate of 0.5 ml/min. The wavelength of the UV detector was set to 220 nm. The standards used in the MW calibration curve were myoglobin (MW 17600 Da), lysozyme (MW 14400 Da), insulin (MW 5800 Da), and vitamin B_12_ (MW 1300 Da). Regression analysis was executed based on the logarithm of the MW of the standards and retention time. Then, the MW of the samples was calculated according to the retention time and regression equation.

### 2.11 Mass spectrometry analysis of the digestion product

The end product of gastrointestinal digestion was selected for mass spectrometry analysis. Experiments were performed on a Q‐Exactive mass spectrometer coupled to Easy nLC (Thermo Fisher Scientific). The peptide mixture was loaded onto a RP‐C18 column (15 cm × 75 μm) packed in‐house with RP‐C18 5 μm resin in buffer A (0.1% formic acid in HPLC‐grade water), and separated with a linear gradient of buffer B (0.1% formic acid in 84% acetonitrile) at a flow rate of 250 nl/min over 60 min. MS data were acquired using a data‐dependent top 10 method, which dynamically chose the most abundant precursor ions from the survey scan (300–1800 m/z) for high energy collision dissociation (HCD) fragmentation. MS data were analyzed using MaxQuant version 1.3.0.5, and searched against the UniProtKB Triticum aestivum database. The search followed an enzymatic cleavage rule of none and mass tolerance of 20 ppm for fragment ions. The cutoff of the global false discovery rate (FDR) for peptide and protein identification was set to 0.01.

### 2.12 Statistical analysis

The experimental results were expressed as mean±standard deviation (*n* = 3). SPSS 22.0 (SPSS Inc., Chicago, IL, USA) was used for significance analysis. The experimental results differed significantly as *p* < .05 (marked with different letters). Origin 8.0 software was used for mapping.

## 3 RESULTS AND DISCUSSION

### 3.1 In vitro digestibility analysis

**FIGURE 1:**
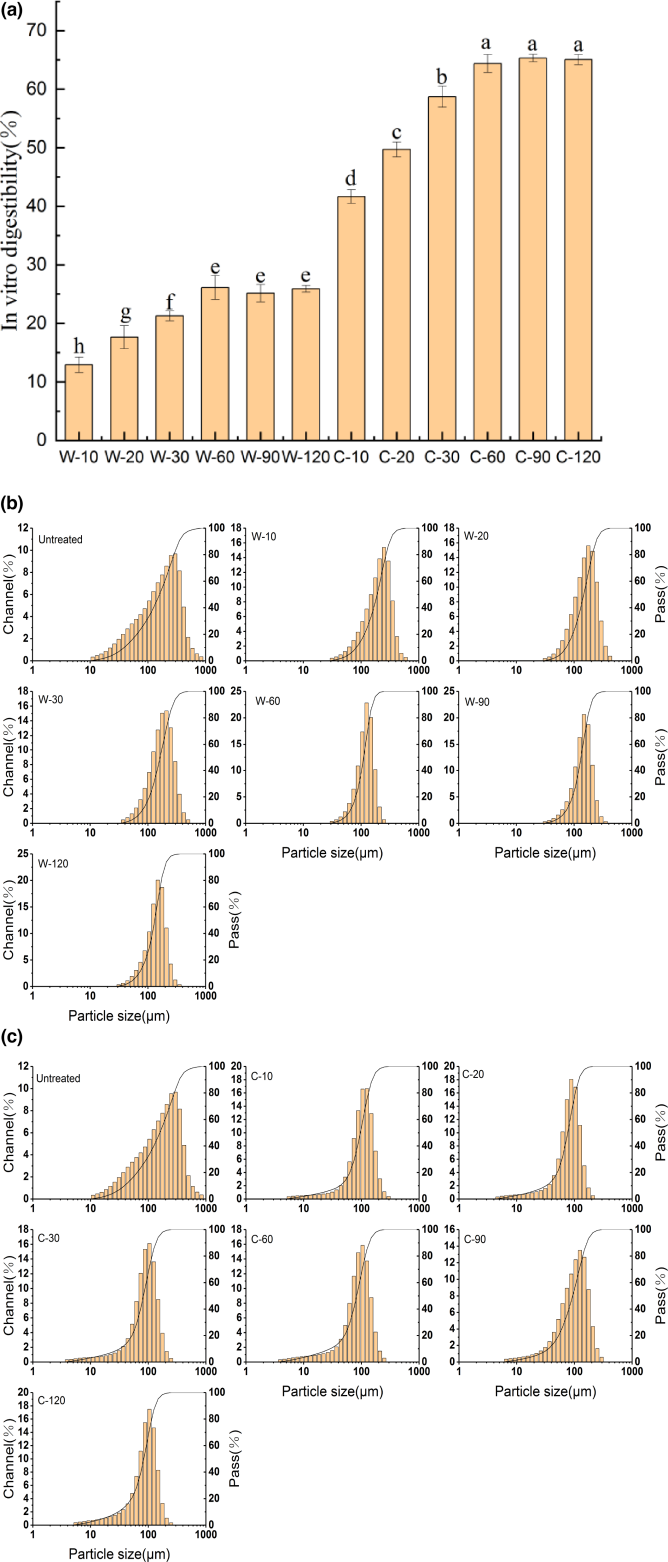
In vitro digestibility of GWP after pepsin and trypsin digestion at different time (a); particle size distribution of pepsin digestion products (b)/pepsin and trypsin digestion products (c) at different times

The digestive property of protein was an important indicator to measure the nutritional value of protein. The in vitro digestibility of GWP after pepsin digestion at different times is shown in Figure 1a. With the increase in the pepsin digestion time from 0 to 60 min, the in vitro digestibility of GWP increased greatly (*p* < .05). The maximum digestibility was 26.12% at 60 min. There was no significant change in digestibility when pepsin digestion time exceeded 60 min (*p* > .05). The reason was that the substrate concentration gradually decreased, and the enzyme reaction sites were gradually saturated in the digestion process. At the same time, the concentration of digestive products increased and their competitive inhibition became stronger (Xka et al., 2007). In the process of simulating digestion in intestine with trypsin, in vitro digestibility increased from 26.12% to 65.32%. The maximum digestibility was also obtained at 60 min of trypsinization. Similar to the digestion process of pepsin, the digestibility remained unchanged after 60 min. The digestibility of mature wheat protein reported in the literature was 49.10% after simulated in vitro digestion with pepsin and trypsin (Abdel‐Aal, 2008). It could be found that GWP was easier to be digested than mature wheat protein. The digestibility of GWP and mature wheat protein was not high, which might be related to low solubility of protein (Căpriţă et al., 2012). Moreover, it could be detected that the digestibility of GWP at the pepsin digestion stage was limited, and the digestibility increased drastically in trypsin digestion. The limited digestibility might be attributed to some amino acid residues such as proline and hydroxyproline in the structure of GWP that resisted pepsin digestion (Udenigwe et al., 2021). The structure of GWP unfolded after pepsin digestion, exposing more trypsin restriction sites, which were more conducive to the digestion of trypsin (Lassé et al., 2015).

### 3.2 Particle size distribution

In order to further characterize the digestion process of GWP, particle sizes of the digestion products at different digestion times were measured. The results were shown in Figure 1b and Table 2, the particle sizes distribution narrowed significantly with the extension of the pepsin digestion time. This implied that pepsin digestion caused more uniform particle size distribution. Compared with undigested GWP, the average particle size (D_50_) of the pepsin digestion products was significantly reduced. In addition, it could be clearly observed that large‐size particles around 1000 μm were gradually decreased. Through further calculations, large‐sized particles (D_90_) and medium‐sized particles (D_50_) were, respectively, reduced by 31.35% and 18.00% after pepsin digestion. The above results revealed that larger size particles in GWP were mainly digested at the simulated gastric digestion stage. It was also proved that pepsin was more suitable for larger particle digestion (Li et al., 2017).

**TABLE 2:** Particle sizes of GWP‐simulated digestion product at different times

Sample	D_50_/μm	D_90_/μm
GWP	191.80 ± 1.90^a^	311.65 ± 0.05^a^
W‐10	184.35 ± 5.05^b^	299.00 ± 4.9^b^
W‐20	178.50 ± 3.10^c^	268.45 ± 0.65^c^
W‐30	169.05 ± 2.25^d^	233.00 ± 13.5^d^
W‐60	159.85 ± 1.65^e^	218.95 ± 4.55^e^
W‐90	158.60 ± 2.40^e^	216.85 ± 6.45^e^
W‐120	157.65 ± 2.15^e^	213.95 ± 3.90^e^
C‐10	128.25 ± 2.35^f^	159.85 ± 2.75^f^
C‐20	116.01 ± 2.86^g^	154.55 ± 1.35^g^
C‐30	95.38 ± 1.67^h^	137.90 ± 2.60^h^
C‐60	86.84 ± 1.58^i^	136.45 ± 2.05^h^
C‐90	88.98 ± 0.96^i^	128.60 ± 2.40^i^
C‐120	90.93 ± 0.82^j^	128.07 ± 1.35^i^

*Note*: Values were expressed as means (*n* = 3) ± *SD*. W‐10, W‐20, W‐30, W‐60, W‐90, and W‐120 indicate pepsin digestion for 10, 20, 30, 60, 90, and 120 min, respectively. C‐10, C‐20, C‐30, C‐60, C‐90, and C‐120 indicate pepsin and trypsin digestion for 10, 20, 30, 60, 90, and 120 min, respectively. Means with different superscripts (a, b) in the same column were statistically different (*p* < .05).

Abbreviation: *SD*, standard deviation.

The particle size distribution of pepsin and trypsin digestion products was shown in Figure 1c. The result showed that the particle size of pepsin and trypsin digestion products was mainly concentrated around 100 μm. Additionally, some substances with a particle size of less than 10 μm appeared in the simulated intestinal digestion products. In the process of simulated intestinal digestion, D_90_ and D_50_ were reduced by 40.14% and 44.78%, respectively (shown in Table 2). This implied that trypsin mainly digested medium‐sized particles into small particles. Furthermore, the D_50_ value was the smallest at 60 min of trypsin digestion. This result was the same as the result of digestibility, which did not change significantly after 60 min of trypsin digestion. Interestingly, the average particle size tended to increase between 60 and 120 min of trypsin digestion. This might be caused by the fact that a smaller particle size resulted in higher surface activity, leading to the phenomenon of particle aggregation (Ye et al., 2010). A similar phenomenon was reported that particle size of both types of milk showed an initial decrease, and then an increase throughout the digestion process (Sun et al., 2019).

### 3.3 SEM analysis

**FIGURE 2:**
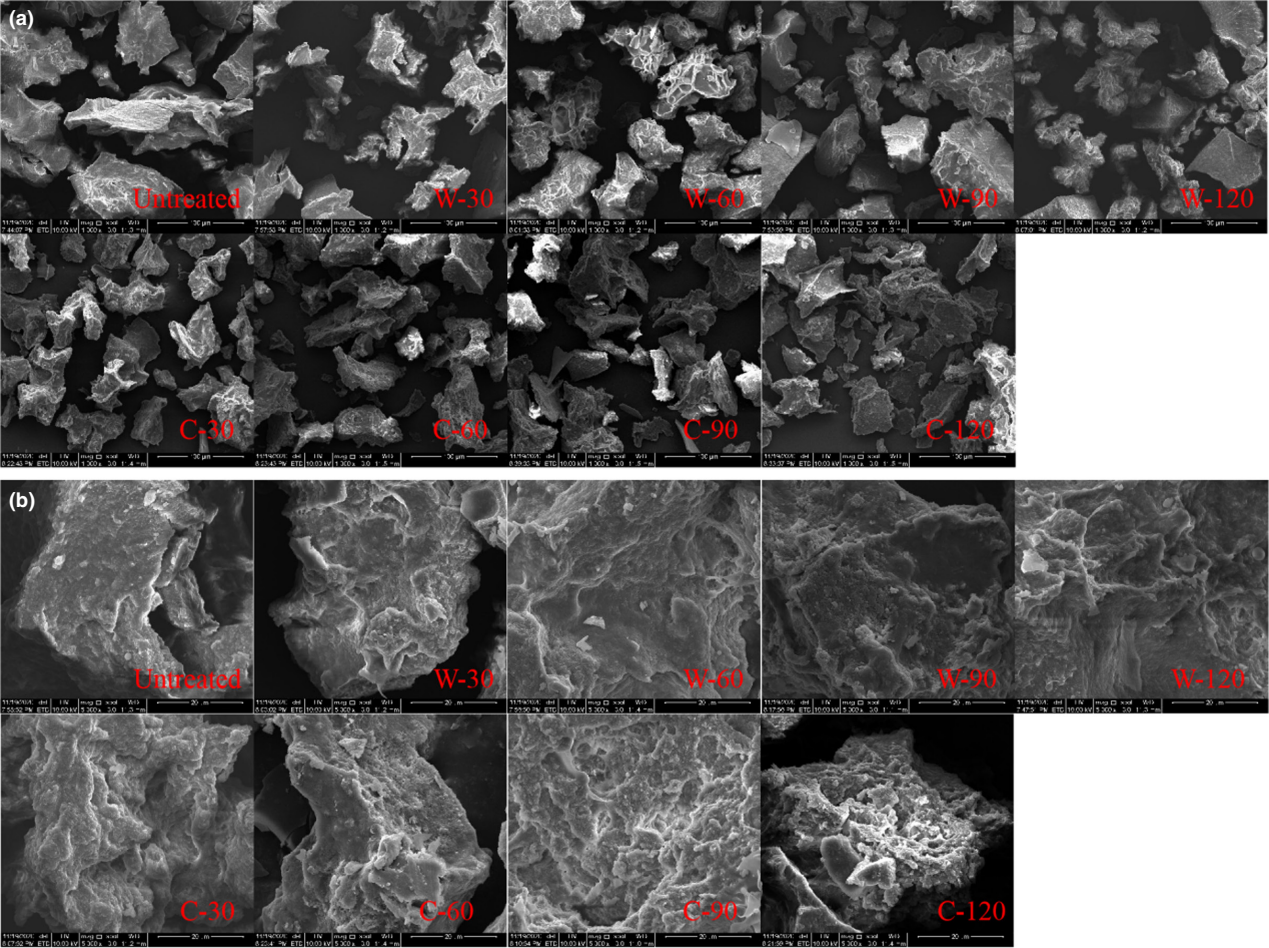
SEM of GWP digested by pepsin and trypsin at a different time under 1000 times magnification (a); SEM of GWP digested by pepsin and trypsin at a different time under 5000 times magnification (b)

Scanning electron microscopy (SEM) was performed to evaluate the effect of simulated digestion on the microstructure of GWP. As shown in Figure 2a, changes in the microstructure could be clearly observed at 1000× magnification. Undigested GWP displayed an irregular large block structure. After pepsin digestion, the larger block structures were reduced, and relatively small block structures appeared. After trypsin digestion was continued, the relatively small block structures were digested into smaller blocks, and smaller block structures gradually increased. This phenomenon was similar to the results of particle size. After the SEM was magnified to 5000 times, the surface changes in block structure were observed in Figure 2b. Obvious cracks and depressions gradually appeared on the smooth surface of the block structure after pepsin digestion. Moreover, there were obvious holes continuing trypsin digestion. This might be a result of GWP structure depolymerization caused by proteolysis (Fei et al., 2010). A similar result was reported in the microstructure changes in tartary buckwheat protein after digestion (Guo et al., 2007)

### 3.4 Intrinsic fluorescence spectroscopy analysis

**FIGURE 3:**
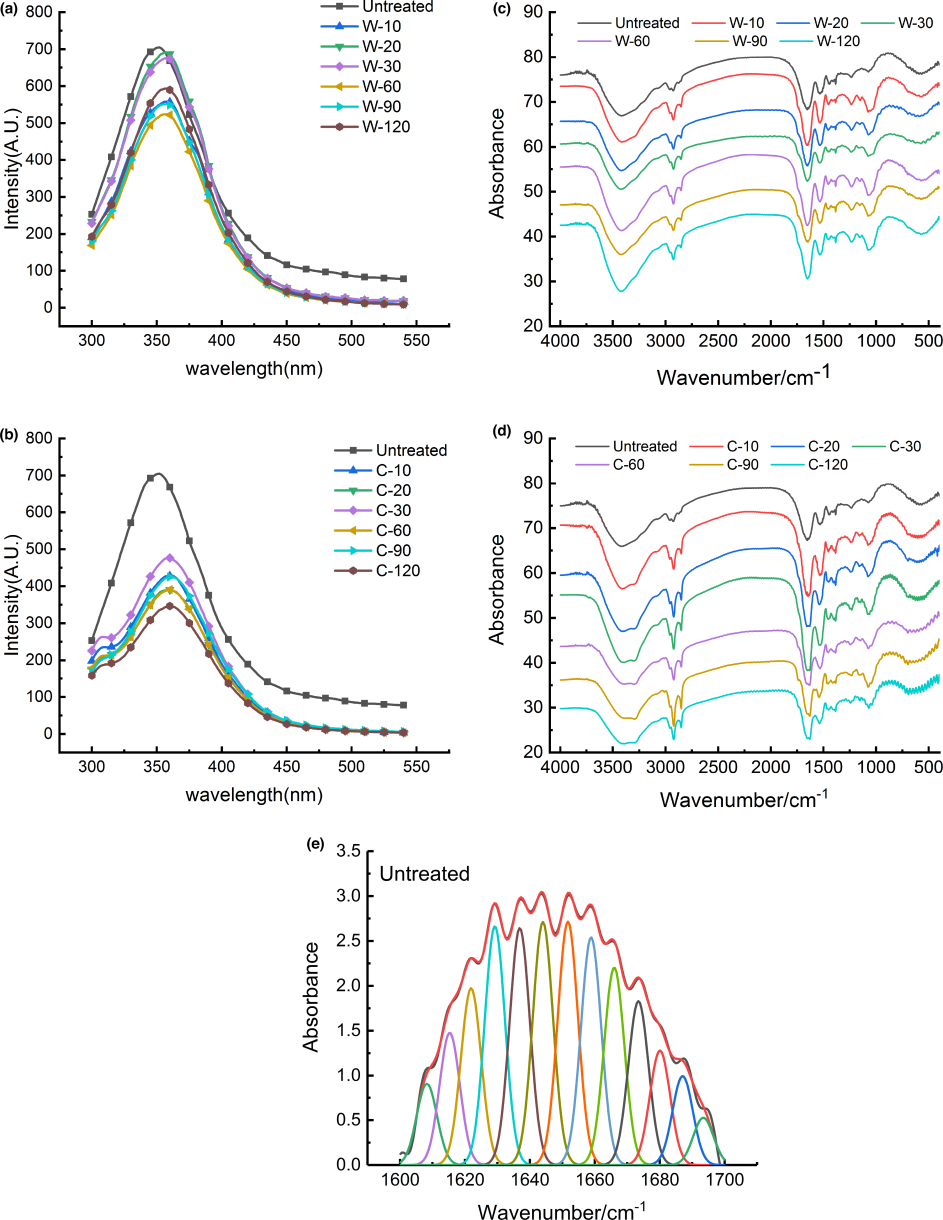
Intrinsic fluorescence spectroscopy of GWP digested by pepsin (a)/pepsin and trypsin (b) at different time; FTIR spectroscopy of GWP digested by pepsin (c)/pepsin and trypsin (d) at different time; and deconvoluted FTIR spectra of GWP (e)

Intrinsic fluorescence spectroscopy was mainly generated by the emission of side chains of tyrosine (Tyr) and tryptophan (Trp) residues in protein at specific wavelengths. Changes in the side chains of protein could be illustrated by fluorescence spectroscopy. The intrinsic fluorescence spectra of digestion samples were shown in Figure 3a,b. The GWP showed a typical intrinsic fluorescence spectrum with the maximum emission wavelength (λ_max_) of 352 nm. After pepsin and trypsin digestion, the fluorescence intensity of GWP decreased significantly, accompanied by a red shift in λ_max_. A similar result also appeared in the fluorescence spectrum of peanut protein isolate after enzymatic hydrolysis (Zhao et al., 2011). During the pepsin digestion process, λ_max_ was red shifted by 6 nm. There was no remarkable change in λ_max_ after 60 min. A continuous red shift was observable during the trypsinization time from 0 to 60 min. Finally, λ_max_ of the digested products was red shifted by 10 nm after pepsin and trypsin digestion. This indicated pepsin and trypsin digestion led to the breakdown of the protein chain and release of peptides. Consequently, more aromatic amino acid residues, buried inside, were exposed to the solvent and GWP was unfolded by enzyme treatment (Albani, 2013). Previous studies also found that λ_max_ had a significant red shift in the detection of intrinsic fluorescence spectra of peanut protein hydrolyzed by enzymes (Zhao et al., 2011).

### 3.5 FTIR spectroscopy analysis

The results of FTIR spectroscopy of GWP during in vitro digestion were displayed in Figure 3c,d. Amide I band (1600–1700 cm^−1^) was the most sensitive to changes in the protein secondary structure (López‐Lorente & Mizaikoff, 2016). Therefore, amide I band was chosen for further analysis. The peak fitting spectrum of GWP was obtained by deconvolution of amide I band, as shown in Figure 3e. To quantitatively measure the contents of the secondary structure components according to the fitting result, the varieties of the secondary structure component contents during the digestion process were listed in Table 3.

**TABLE 3:** The secondary structure content of GWP during the pepsin and trypsin digestion process calculated from FTIR measurements

Samples	Secondary structure composition (%)			
	α‐Helix	β‐Sheet	β‐Turn	Random coil
GWP	23.61 ± 0.06^a^	53.54 ± 0.50^a^	11.92 ± 0.04^a^	10.94 ± 0.52^a^
W‐10	24.06 ± 0.54^a^	51.80 ± 0.12^b^	12.10 ± 0.15^ab^	12.06 ± 0.52^bc^
W‐20	24.97 ± 0.08^b^	50.64 ± 0.13^c^	12.25 ± 0.11^ab^	12.14 ± 0.11^bcd^
W‐30	25.19 ± 0.15^b^	50.26 ± 0.94^cd^	12.06 ± 0.49^ab^	12.49 ± 0.29^cde^
W‐60	25.46 ± 0.12^c^	49.82 ± 0.62^cd^	12.22 ± 0.16^ab^	12.51 ± 0.16^cde^
W‐90	25.49 ± 0.27^c^	49.95 ± 0.60^cd^	11.83 ± 0.18^a^	12.74 ± 0.17^def^
W‐120	25.42 ± 0.64^c^	49.48 ± 0.19^d^	12.28 ± 0.33^ab^	12.83 ± 0.23^efg^
C‐10	25.45 ± 0.64^c^	48.01 ± 0.12^e^	13.27 ± 0.13^abc^	13.28 ± 0.11^fgh^
C‐20	25.48 ± 0.17^c^	47.80 ± 0.15^e^	13.30 ± 0.69^c^	13.42 ± 0.23^hk^
C‐30	25.49 ± 0.65^c^	47.41 ± 0.21^f^	13.50 ± 0.01^bc^	13.61 ± 0.13^hk^
C‐60	26.55 ± 0.24^d^	46.59 ± 0.52^g^	12.86 ± 1.02^abc^	14.01 ± 0.41^k^
C‐90	26.59 ± 0.96^d^	46.58 ± 0.16^g^	13.27 ± 1.27^abc^	13.56 ± 0.16^hk^
C‐120	26.61 ± 0.43^d^	47.09 ± 0.17^g^	12.93 ± 0.69^abc^	13.38 ± 0.10^gh^

*Note*: Values were expressed as means (*n* = 3) ± *SD*. Means with different superscripts (a, b) in the same column were statistically different (*p* < .05).

Abbreviation: *SD*, standard deviation.

According to the literature, bands at 1650–1660 cm^−1^, 1618–1640 cm^−1^ and 1670–1690 cm^−1^, 1660–1670 cm^−1^ and 1690–1700 cm^−1^ and 1640–1650 cm^−1^ were assigned to α‐helix, β‐sheet, β‐turn and random coils, respectively (Wang et al., 2014). The calculation results showed that contents of α‐helix, β‐sheet, β‐turn, and random coils of GWP were 23.6%, 53.5%, 11.9%, and 10.9%, respectively. This indicated that β‐sheet was the main structure of GWP, which was the same as the main structure of wheat protein. Compared with the contents of the secondary structure components of wheat protein reported by Wang et al. (2016), the content of β‐sheet in GWP was markedly higher, while the contents of β‐turn and random coils were less in evidence.

After pepsin digestion, the secondary structure of GWP changed remarkably (*p* < .05). In the first 60 min of pepsin digestion, β‐sheet content decreased from 53.54% to 49.82%. The contents of α‐helix and random coils displayed an obvious upward trend (*p* < .05). The subsequent trypsin digestion results showed the same variation trend. As could be seen from the process of trypsin digestion, the β‐sheet content was reduced by 2.4%, reaching the minimum at 60 min of digestion, while the contents of α‐helix and random coil increased dramatically. Zhao et al. (2013) also found that the β‐sheet content decreased significantly during soybean proteolysis, but the α‐helix content increased observably.

The total content of ordered structures (α‐helix and β‐sheet) of GMP was remarkably reduced during the entire digestion process. Among them, β‐sheet was mainly damaged, which was reduced by 6.28% in total. This result indicated that the structure of GWP was loosened during the digestion of pepsin and trypsin mainly by the unfolding of β‐sheet structure. The experimental results of intrinsic fluorescence spectroscopy also proved that the structure of GWP was unfolded. The secondary structure of rice glutelin (RG) in the digestion process also showed similar changes. The β‐sheet content of RG was obviously reduced, and enzyme hydrolysis led to a more extended secondary structure of RG (Xu et al., 2016).

### 3.6 SDS‐PAGE analysis

**FIGURE 4:**
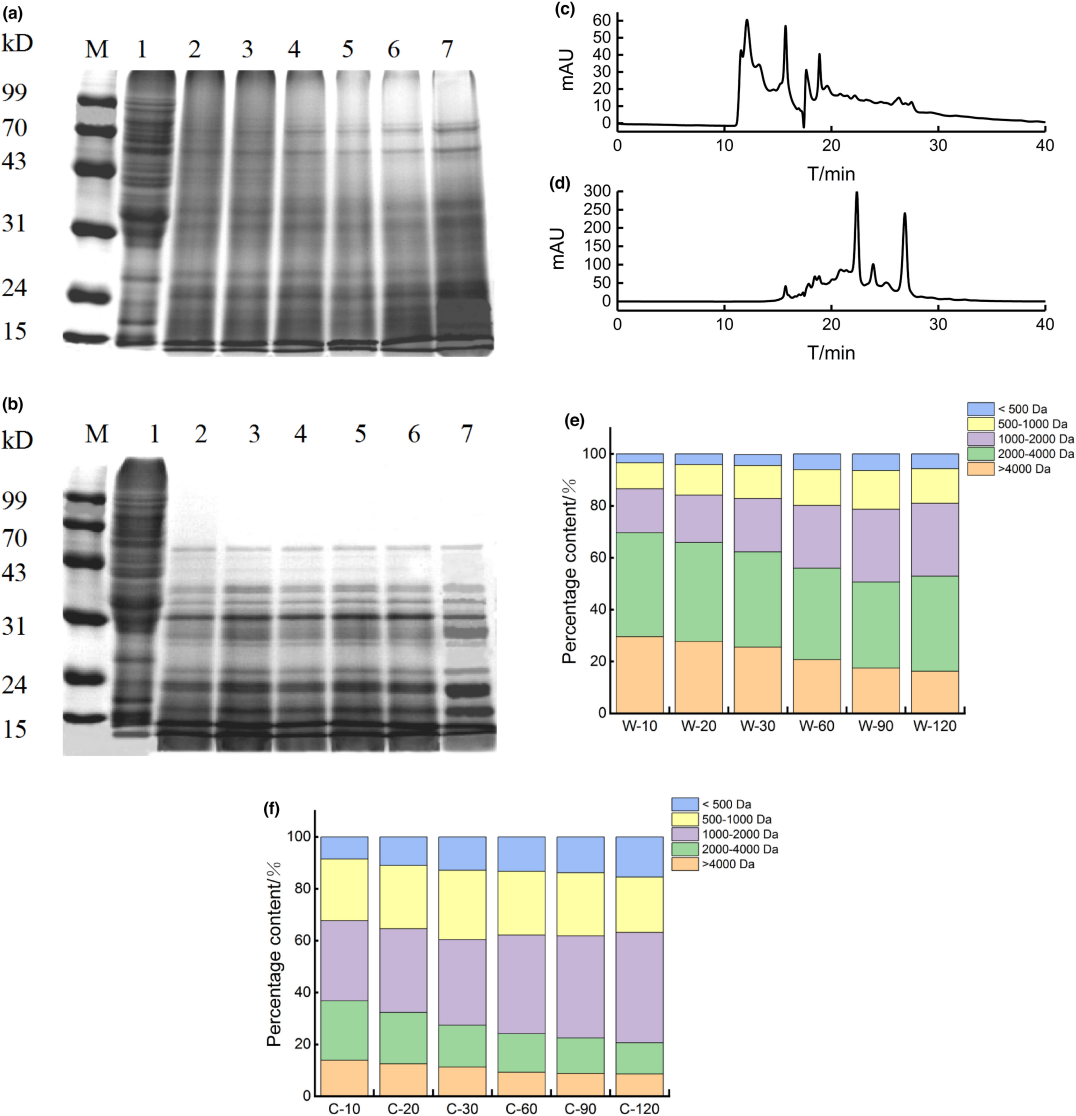
SDS‐PAGE profiles of GWP digested by pepsin (a)/pepsin and trypsin (b) at different times, M: Maker; lane 1: GWP; lane 2–7: pepsin/pepsin and trypsin digestion for 10, 20, 30, 60, 90, and 120 min. Elution profiles of GWP were obtained with pepsin (c)/pepsin and trypsin (d) at 120 min, respectively. MW distribution of GWP digested by pepsin (e)/pepsin and trypsin (f) at a different time

To further characterize the changes in subunit composition of GWP during digestion, digestion products were subjected to SDS‐PAGE experiments. The electrophoretic profiles of samples were shown in Figure 4a. Compared to undigested GWP, the electrophoresis bands of digested products around 90 kDa were dramatically reduced during pepsin digestion. Additionally, bands near 70 kDa and 43 kDa were also gradually reduced, whereas bands near 31 kDa and 24 kDa were obviously increased. The molecular weight (MW) of 70–90 kDa was mainly high‐molecular‐weight (HMW) glutenin, and the MW of low‐molecular‐weight (LMW) glutenin was mainly concentrated in 34–54 kDa (Luo et al., 2001). Moreover, there were four main types of gliadin: α/β‐gliadins (MW 28–35 kDa), γ‐gliadins (MW 35 kDa), ω1,2‐gliadins (45 kDa), and ω‐5gliadins (MW 55–60 kDa), respectively (Rahaman et al., 2016). This result suggested that HMW glutenin and part of LMW glutenin and ω‐gliadin were mainly digested by pepsin.

The results of SDS‐PAGE by trypsin digestion at different times were displayed in Figure 4b. Bands with relative MW above 50 kDa disappeared at 20 min of trypsin digestion. In addition, there were fewer bands with a MW about 43 kDa and 31 kDa, while more bands around 24 kDa and 15 kDa in trypsin digestion products compared with pepsin digestion products. These suggested that medium‐sized MW subunits were broken into smaller peptides. This result was also proved by the particle size experiment in which larger‐sized particles mainly were digested by pepsin, while medium‐sized particles were digested by trypsin. Kong et al. (2007) also found a similar result. The electrophoresis pattern of wheat protein digested with trypsin showed that large polypeptides above 30 kDa decreased significantly, while small peptides close to 10 kDa increased drastically. In summary, it could be found that HMW glutenin and ω‐gliadins were completely digested in the gastrointestinal digestion process of GWP, while LMW glutenin and α/β/γ‐gliadins were partially digested.

### 3.7 Molecular weight distribution analysis

The molecular weight distributions of the final digestion product of GWP by pepsin and trypsin were shown in the Figure 4c,d. It could be clearly found that there were significant differences in the molecular weight distribution of pepsin and trypsin digestion products. Therefore, a specific analysis of MW composition in digestion products was carried out, and the results were shown in Figure 4e,f. Peptides with MW above 2000 Da were the main components in the products of pepsin digestion, and it still accounted for 52.9% after 120 min of pepsin digestion. In the process of pepsin digestion, the MW above 4000 Da decreased by 13.38%, mainly converted into 1000–2000 Da peptides, which increased by 11.14%. In the trypsin digestion stage, peptides with a MW of 2000–4000 Da and greater than 4000 Da were reduced by 10.92% and 4.28%, and peptides with a MW of 1000–2000 Da and less than 500 Da increased by 11.71% and 6.94%, respectively. Additionally, obviously different from the pepsin digestion products, the MW of trypsin digestion products mainly fell into the range 500–2000 Da, and it accounted for 63.9% of the final digestion products. The research of Zheng et al (Zheng et al., 2017) also found that the MW of enzymatic hydrolysis products of wheat protein by trypsin was mainly concentrated below 2000 Da.

### 3.8 Peptide profiling of GWP digestion products

**FIGURE 5:**
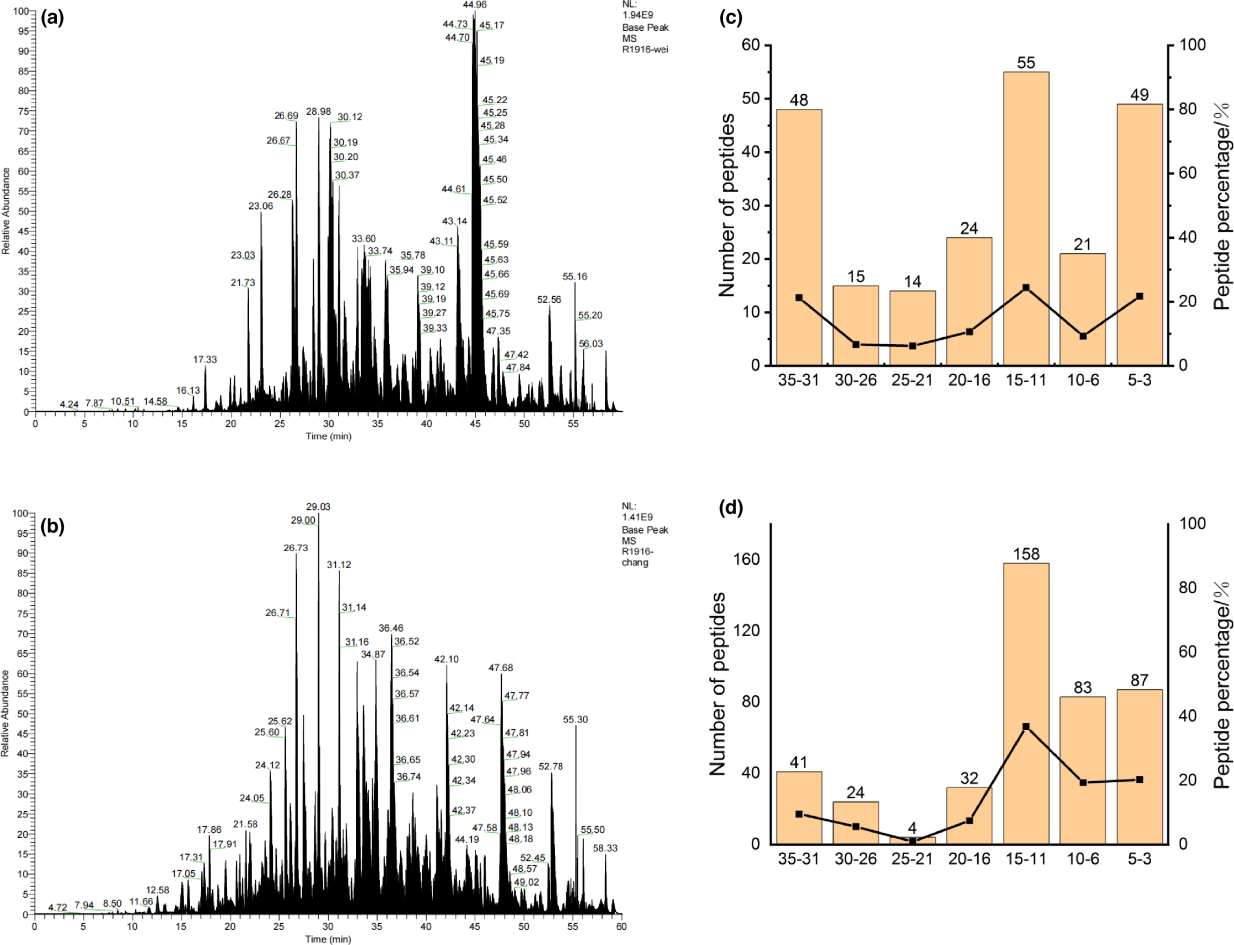
Total ion current chromatograms of digestion products of GWP by pepsin (a)/pepsin and trypsin (b); peptide length distribution in digestion products of GWP by pepsin (c)/pepsin and trypsin (d)

In this study, the final products of simulated stomach and intestine digestion were analyzed by using LC‐MS to determine the composition of peptides. The total ion current chromatograms of the two digested products were shown in Figure 5a,b. It was clear that the total ion current chromatograms of the two digestion products were apparently different. The LC‐MS results were further analyzed, the numbers of peptides obtained from GWP after pepsin digestion and trypsin digestion were 186 and 429 peptides, respectively. This result suggested more proteins were digested to fragments during trypsin digestion, which was in accordance with the result of digestibility.

These peptides were then sequenced, the length distribution of the collected peptides was analyzed, and the results were displayed in Figure 5c,d. The peptide length of 3–35 amino acids was searched by Maxquant software. The database search results showed that the peptide length distribution was relatively scattered after pepsin digestion. Peptides with the lengths of 11–15, 31–35, and 3–5 amino acids accounted for 24.3%, 21.2%, and 21.7% of the total peptides, respectively. However, the peptide length of the digested products was mainly concentrated in 3–15 amino acid residues after further digestion by trypsin. The number of peptides with the length of 3–15 amino acids accounted for 76.5% of the total peptides. What is more, compared with simulated stomach digestion products, there were relatively fewer peptides with 16–35 amino acids after trypsin digestion. Among them, the number of peptides with 31–35 amino acids decreased the most, which accounted for 11.7%. In contrast, the number of peptides with 6–15 amino acids increased the most. Therefore, GWP was first digested by pepsin for multisite digestion, and then trypsin was mainly used to digest peptides of 16–35 amino acids into short peptides of 6–15 amino acids.

As we all know, wheat protein was closely related to coeliac disease. It was an autoimmune enteropathy that occurred in genetically predisposed subjects after ingestion of gluten or related protein (Kagnoff, 2007). The immunogenic peptides in wheat protein related to coeliac disease in the known literature were as follows: TQQPQQPFPQ, SQQPQQPFPQPQ, TQQPQQPFPQQPQQPFPQ, PQTQQPQQPFPQFQQPQQPFPQPQQP, PFPQPQQPQQPFPQSQQPQQPFPQP, PQQPQLPFPQQPQQPFPQPQQPQ, and QQPQQPFPQPQQTFPQQPQLPFPQQPQQPFP (Prandi et al., 2017). The above allergenic peptide sequence was not detected in the digested products of GWP by pepsin. However, QPQQPFPQQPQQPSPQTQQPQQPFPQQPQQP, PQQPQQPFPPPQQPQQPFPQSQQPQQPFPQPQQP, PQQPQQPFPQPQQPQQPFSQSQQPQQPFPQPQQP, and FPQQPQLPFPQQPQQPFPQPQQPQQPFPQ were found in the samples obtained after two‐step digestion of GWP with pepsin and trypsin. Therefore, GWP was still unsafe for patients with coeliac disease, and further tests on the contents of allergenic peptides contained in GWP were needed.

## 4 CONCLUSIONS

GWP’s, as a fresh cereal protein, digestive properties were closely related to its application. By simulating the digestive environment, this study clearly revealed the changes in structure, particle size, MW, and digestion product composition of GWP during the digestion process. The results showed that digestibility of GWP was 65.32% after pepsin and trypsin digestion. HMW glutenin and ω‐gliadins in GWP were completely digested, while LMW glutenin and α/β/γ‐gliadins were partially digested. Furthermore, the MW of pepsin digestion products was mainly above 2000 Da, while the MW of most trypsin digestion products was 500–2000 Da. Additionally, immunogenic peptides associated with coeliac disease were detected after GWP was digested with pepsin and trypsin. In the future, allergenic peptides of GWP need to be further studied. This research provides a theoretical basis for expanding the application of green wheat, and characterizes the digestion properties of protein in green wheat which is the main nutrient. Previous studies only focused on the quality and application of green wheat, but paid little attention to nutrients. In addition, it also provides a high‐quality grain substrate for the plant‐based food industry.

## CONFLICTS OF INTEREST

The authors claim no conflict of interests in this work.

## Data Availability

The data that support the findings of this study are available from the corresponding author upon reasonable request.
